# Causal Relationship Between Genetically Determined Free Fatty Acids and Nonalcoholic Fatty Liver Disease: A Mendelian Randomization Study

**DOI:** 10.1155/ije/2181771

**Published:** 2025-10-07

**Authors:** Kenan Li, Yuyin Liang, Xiumin Zhang, Piao Hu, Manyi Hu

**Affiliations:** ^1^Department of Infectious Diseases, Xiaoshan Affiliated Hospital of Wenzhou Medical University, Hangzhou, Zhejiang, China; ^2^Department of Infectious Diseases, The Fifth Affiliated Hospital, Sun Yat-Sen University, Zhuhai, Guangdong, China; ^3^Department of Refractive Clinic, Hangzhou Aier Eye Hospital, Hangzhou, Zhejiang, China; ^4^Department of Colorectal Surgery, Jinhua Municipal Central Hospital, Jinhua, Zhejiang, China

**Keywords:** causality, FFAs, Mendelian randomization analysis, NAFLD

## Abstract

**Objective:**

This study aimed to evaluate whether the ten-individual plasma-level of free fatty acids (FFAs) affect the risk of nonalcoholic fatty liver disease (NAFLD) which is featured by triglycerides (TGs) deposition because the observational studies are limited and conflicting about the causal effect between FFAs and NAFLD.

**Design and Methods:**

This analysis was a large genome-wide association study (GWAS) summary statistic. Fourteen independent single-nucleotide polymorphisms (SNPs) without linkage disequilibrium (*r*^2^ < 0.005) that were strongly associated (*p* < 5 × 10^−8^) with FFAs were chosen as instrumental variables to estimate the causal effect of genetic variants on the levels of 10 phospholipid FAs through GWAS, and summary estimates were obtained using the inverse-variance weighted (IVW) method applied to SNPs. And the summary-level data of European participants from the eMERGE network, the FinnGen cohorts, the UK Biobank, and the Estonian Biobank, for the latest and largest GWAS datasets for NAFLD (8434 cases and 770,180 controls), were obtained. Mendelian randomization analysis was applied.

**Results:**

The result demonstrated that 10 individual FFAs were not significantly associated with NAFLD.

**Conclusion:**

The evidence to support the causal association of the individual plasma FFAs with NAFLD is insufficient in this study.

## 1. Introduction

Nonalcoholic fatty liver disease (NAFLD) is a liver disease with a metabolic disorder featured by more than 5% hepatocytes of triglycerides (TGs) deposition, and it is apart from alcohol consumption, drugs, toxins, or infectious induced liver disease [1]. Additionally, NAFLD has been recognized as the most common liver disorder worldwide since the late 20th century. It is estimated that NAFLD affects up to 25% of the world's population, and people with risk factors are as high as 60%–80% [2, 3]. The progression of NAFLD is from steatosis of hepatocytes to nonalcoholic steatohepatitis (NASH) which is contributed by inflammatory fibrosis, and then cirrhosis. Serious complications such as abdominal obesity, T2DM, hypertension, and atherogenic dyslipidemia of NAFLD are quite high in prevalence, and patients with fibrosis Stages 3–4 irrespective of NASH predicted the increasing mortality [4].

In recent years, some experts have proposed using metabolic associated fatty liver disease (MAFLD) to rename NAFLD [5, 6], which emphasizes the metabolic syndrome of the liver and the cause of excessive hepatic lipid deposition (steatosis). Ingestion of dietary lipids is considered to contribute to the pathogenesis of NAFLD by elevating levels of free fatty acids (FFAs) in the systemic circulation [7–10]. Increased concentrations of serum lipids can induce aberrant accumulation of lipids within tissues like the liver and heart, thereby promoting the development of atherosclerosis and NAFLD [11]. And the faster pace of life and industrial food production has led to increased intake of fats, especially saturated fat and cholesterol. These fats in the state of FFAs can contribute to insulin resistance and inflammation, which can increase the risk of heart disease, stroke, and type 2 diabetes [12–14]. Hepatic lipids can originate from the diet or be released from peripheral adipose tissue.

Lacking biomarkers makes it hard to diagnose clinically, and efficacy is impaired by ineffective patient stratification of the complex progression spectrum of NAFLD [15]; the development of novel therapeutic and preventative strategies to attenuate the expansion of NAFLD is of great necessity [16, 17]. Roles of fatty acids on NAFLD have always been a focus for researchers, there is a growing body of evidence that suggests that FFAs play a key role in the pathogenesis of NAFLD [18–20], and previous studies showed that specific FA and their ratios may suit to be as biomarkers for NASH [21], a severe progression of NAFLD, while the causal evidence between FFAs and NAFLD is still in a great deal of ambiguous despite extensive attempts [22–24].

NAFLD, like many chronic conditions, exhibits a complex and multifaceted etiology. This complexity makes it challenging to definitively establish causal relationships between risk factors (exposures) and disease outcomes. Unlike randomized controlled trials (RCTs), which are often infeasible due to ethical considerations, Mendelian randomization (MR) analysis [25] offers a robust approach to investigate causality. MR methods [26] leverage genetic variants identified through GWAS [27] as instrumental variables (IVs) [28] to estimate causal relationships between exposures and outcomes. This approach mimics the randomization of a large clinical trial and thereby mitigates confounding effects, a major limitation of observational cohort studies. In this study, we employed MR analysis to leverage human genetic data to estimate the causal effects of 10 fatty acids (FAs): 6 polyunsaturated fatty acids (PUFAs), 2 monounsaturated fatty acids (MUFAs), and 2 saturated fatty acids (SFAs) on NAFLD risk.

## 2. Materials and Methods

### 2.1. Study Design and Data Sources

**Figure 1:**
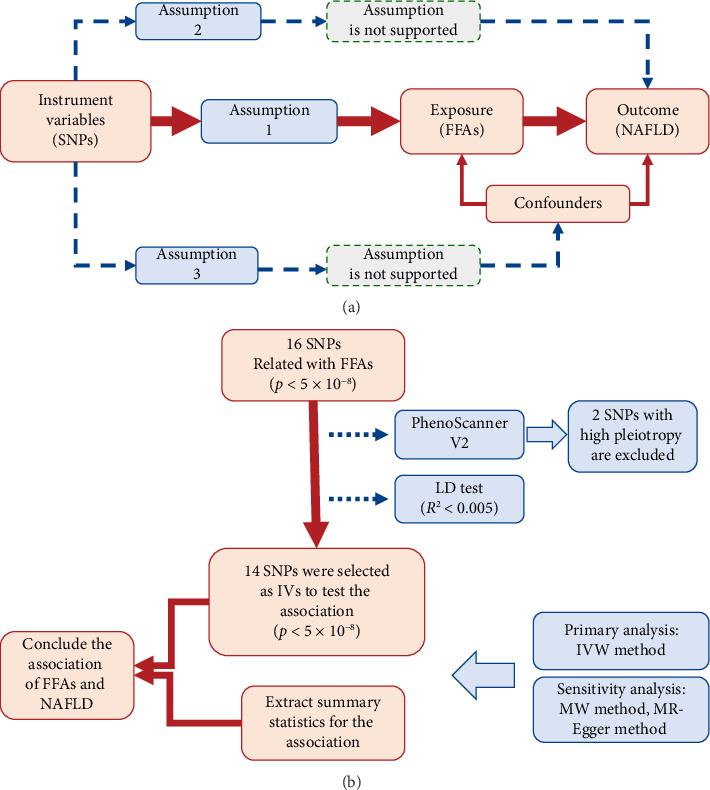
Assumptions and framework of the study design. (a) Three core assumptions about MR analysis; (b) A flowchart about the design of this work. SNPs, single-nucleotide polymorphisms; FFAs, free fatty acids; LD test, linkage disequilibrium test; NAFLD, nonalcoholic fatty liver disease; IVW, inverse-variance weighted; MR-Egger, Mendelian randomization-Egger regression. The dotted line indicates that there is the pleiotropic or direct causal relationship between exposure and outcome.

This investigation employed a two-sample MR framework to assess the causal influence of FFAs on NAFLD. It is essential to follow three core assumptions when MR analysis is designed. In this case of FFAs and NAFLD, genetic variants strongly associated with 10 fatty acids (Assumption 1), getting rid of confounders which may also affect the causal association of exposure (FFAs) and outcome (NAFLD) (Assumption 2), and IVs (single-nucleotide polymorphisms (SNPs)) can only affect NAFLD risk through FFAs (Assumption 3). The MR study design is illustrated in a flowchart format in Figure 1.

The GWASs for NAFLD were conducted in a sizeable European ancestry population with 8434 cases and 770,180 controls [29]. This dataset encompassed participants from four independent studies utilizing electronic health record (EHR) documentation: the Electronic Medical Records and Genomics (eMERGE) network: 9677 participants (1106 cases and 8571 controls); The UK Biobank: an updated NAFLD GWAS analysis included 307,799 participants (2558 cases and 395,241 controls) [30]; The FinnGen cohorts: 176,899 participants were analyzed (651 cases and 176,248 controls). The Estonian Biobank new GWAS: 194,239 participants (4119 cases and 190,120 controls). This study aimed to understand the association of FFAs with outcomes on NAFLD and genetic variants of 14 SNPs from 10 FFAs, and specific information is shown in Table 1. And the entirety of the analytical framework for this study was constructed upon publicly available summary statistics.

### 2.2. Selection of Instrumental Variables

SNPs with genome-wide significant associations (*p* < 5 × 10^−8^) with FFA levels were chosen as IVs. We selected 16 SNPs from the Cohorts for Heart and Aging Research in Genomic Epidemiology (CHARGE) consortium, focusing on individuals of European ancestry, and in detail, SNPs for four n-3 PUFA (ALA, EPA, DPA, and DHA) were obtained from 8866 individuals, SNPs for two n-6 PUFA (LA and AA) and SNPs for one n-7 MUFA(POA) are chosen from 8631 individuals, and one n-9 MUFA(OA) and two SFA (PA and SA) were selected from 8961 individuals. In order to get rid of confounders from Consumption 2 and guarantee that the IVs were not directly associated with NAFLD [31], PhenoScanner V2 analysis was applied, and the two SNPs (rs780093 and rs780094) potentially pleiotropic for the body mass index and alcohol consumption were excluded. To ensure the remaining SNPs acted independently, we assessed their linkage disequilibrium (LD) using the LDlink web-tool (https://ldlink.nci.nih.gov/, Utah Residents from North and West Europe population). This online platform facilitates LD analysis in specific populations. We employed a stringent threshold (*r*^2^ < 0.005) to identify a set of independent SNPs (detailed in Supporting Tables 1 and 2). Ultimately, fourteen genetically independent SNPs were selected as the IVs to estimate serum phospholipid levels of n-3 PUFAs (*n* = 8866 individuals) [32], *n* − 6 PUFAs (*n* = 8631 individuals) [33], and MUFA and SFAs (*n* = 8961 individuals) [34]. The selection of these specific SNPs was informed by previous research [35–38], where their validity and directionality as IVs have been established across various studies.

### 2.3. Statistical Methods

To estimate the causal effect of FFAs on NAFLD, inverse-variance weighted (IVW) MR was employed as the primary analysis. This method leverages validated genetic variants (SNPs) as instrumental variables. By utilizing genetic predisposition as an unconfounded proxy for FFA levels, IVW MR aims to mitigate the influence of confounding factors and generate unbiased estimates of the causal relationship between FFAs and NAFLD. Sensitivity analyses were conducted to evaluate the robustness of the findings and account for potential horizontal pleiotropy. This phenomenon arises when the SNPs not only influence FFA levels but also exert independent effects on other traits that may influence NAFLD development. To address this, the weighted median (WM) and MR-Egger regression (MR-Egger) methods were employed. These methods offer alternative estimations under different assumptions about pleiotropic effects. The WM method offers a robust estimate under the assumption that no more than 50% of the information from the instrumental variables is invalid due to pleiotropy [39]. It summarizes genetic association data across multiple SNPs into a single causal estimate, providing reliable results even in the presence of a moderate degree of pleiotropic effects. MR-Egger views bias from pleiotropy as analogous to small study bias and can detect violations of certain IV assumptions [39–41]. It allows for unbiased causal effect estimates even when pleiotropic effects are present, but with the caveat that the validity of MR-Egger estimates relies on the assumption that the strength of the SNPs' association with the outcome (NAFLD) is independent of the pleiotropic effects. All statistical tests were two-sided, and a *p* value threshold of less than 0.05 was used to determine statistical significance. To account for multiple testing across the 10 fatty acid exposures analyzed, a Bonferroni correction was applied, and statistical significance was defined as a *p* value < 0.005 (0.05/10). The analyses were performed using R software (version 4.2.2) with the “MendelianRandomization” and “TwoSampleMR” packages.

## 3. Results

### 3.1. Association of FFA Variants With NAFLD

**Figure 2:**
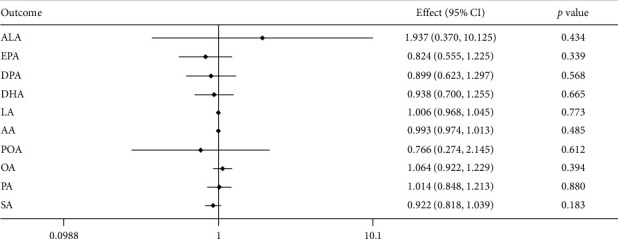
Forest plot of the causal associations of FFAs levels with the risk of nonalcoholic fatty liver disease (NAFLD) in MR analyses. The effects, 95% CIs, and *p* values of associations are contained. Effect, the combined causal effect; CI, confidence interval; *p* value, *p* value of the causal estimate, and the ORs of NAFLD were scaled to a per SD increase in FFA levels.

All the 10 metabolic factors (FFAs) were not significantly associated with NAFLD risk in the combined dataset. The associations between NAFLD and each FFA are shown in Figure 2. For *n* − 3 PUFAs, the odds ratio (OR) of ALA is 1.937, 95% confidence interval (CI) 0.63–1.297 per SD-increase, the OR of EPA is 0.824, 95% CI 0.555–1.225 per SD-increase, the OR of DPA is 0.899, 95% CI 1.48–2.24 per SD-increase, and the OR of DHA is 0.938, 95% CI 0.7–1.255 per SD-increase. For *n* − 6 PUFAs, the OR of LA is 1.006; 95% CI 0.968–1.045 per SD-increase, the OR of AA is 0.993, 95% CI 0.974–1.013 per SD-increase. For *n* − 7 MUFA, the OR of POA is 0.766, 95% CI 0.274–2.145 per SD-increase. For *n* − 9 MUFA, the OR of OA is 1.064, 95% CI 0.992–1.229 per SD-increase. For SFA, the OR of PA is 1.014; 95% CI 0.848–1.213 per SD-increase, and the OR of SA is 0.992; 95% CI 0.818–1.039 per-SD increase. All the *p* values of these FFAs are more than 0.005.

**Figure 3:**
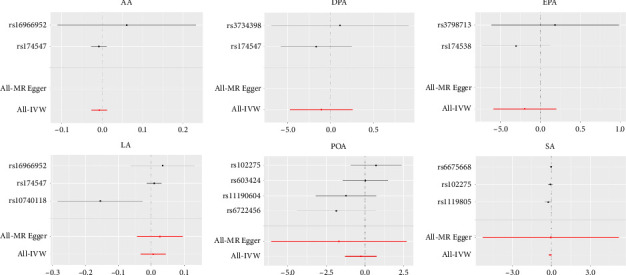
Forest plot depicting the effect of the genetic level of FFAs with the risk of NAFLD in MR analyses by each and all SNPs, using MR Egger and IVW Mendelian randomization. IVW, inverse-variance weighted.

The effects of individual SNP for NAFLD are shown in Figure 3, as the picture shown as follows, where we conduct analysis and separate SNPs in FFAs involving AA, DPA, EPA, LA, POA, and SA, specifically. The results indicate that none of the six FFA individuals are in causal association with NAFLD, and it is consistent with the result in Figure 2.

### 3.2. Sensitivity Analyses

**Figure 4:**
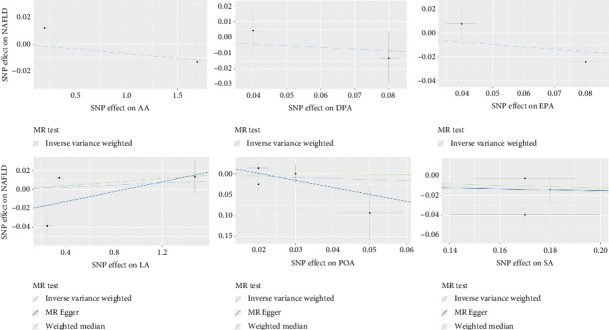
Scatter plots for a single causal effect (AA, EPA, DPA, HDA, LA, POA, and SA) estimates on NAFLD risk.

We employed various approaches, including the WM and MR-Egger methods, to assess the validity of our instrumental variables and investigate potential confounding due to pleiotropy (effects of the genetic variants on outcomes other than NAFLD). The MR-Egger intercept provided no evidence of statistically significant directional pleiotropy (*p* > 0.05) among the instruments, suggesting minimal influence of pleiotropic effects biasing our results. The WM estimates for NAFLD (LA: *β* 0.01, *p*=0.394; SA: *β* −0.062, *p*=0.376; POA: *β* −0.056, *p*=0.926) (Supporting Table S3) were directionally consistent with the primary analysis, further supporting the robustness of our findings. Additionally, MR-Egger regression yielded intercepts close to zero and nonsignificant *p* values (LA: intercept −0.024, *p*=0.454; SA: intercept: 0.007, *p*=0.988; POA: intercept: 0.035, *p*=0.511) (Supporting Table S3), reinforcing the notion that directional pleiotropic effects of these specific fatty acids (LA, SA, and POA) are unlikely to have significantly biased the estimated causal effect on the NAFLD risk. And MR-PRESSO did not denote any pleiotropic SNP, suggesting no bias between POA and NAFLD (Supporting Table S3). Causal effect estimates for NAFLD risk were obtained for each individual genetic variant using 3 MR methods: IVW, WM, and MR-Egger method. Scatter plots were generated to visualize these single causal effect estimates. On the *x*-axis, each point represents the estimated causal effect of a single genetic variant on the FFA level. The *y*-axis depicts the association between FFA levels and NAFLD risk. The horizontal line represents the overall causal effect of FFA levels on NAFLD risk, estimated by combining the information from all genetic variants (Figure 4). The sensitivity analysis demonstrated that our results were stable and reliable. Also, we used the MR-Steiger method to test for reverse causality, and the results showed no evidence of it (Supporting Table S4).

## 4. Discussion

This study conducted MR to delineate potential causal relationships between a comprehensive panel of 10 FFAs (ALA, EPA, DPA, EPA, LA, AA, POA, OA, PA, and SA) and NAFLD. To the best of our knowledge, this represents the inaugural MR inquiry examining such a broad range of FFAs in the context of NAFLD pathogenesis. These findings yield inconclusive evidence to support a causal association between the investigated 10 individual FFAs and NAFLD. This observation appears to diverge from established understandings regarding the potential roles of specific FFAs in NAFLD development. There are discrepant findings and inconsistencies observed in research regarding individual fatty acids impact on NAFLD. Several studies observed an inverse association between dietary *n* − 3 and *n* − 6 PUFA intake and NAFLD risk [42]. [43]. However, contrasting findings reported significantly higher n-6 PUFA intake and *n* − 6/*n* − 3 ratio in NAFLD patients [44]. Lipidomic studies failed to detect significant differences in FFA levels between NAFLD patients and controls [45, 46]. Meta-analyses and clinical trials yielded mixed results regarding the therapeutic effect of *n* − 3 PUFA supplementation on NAFLD [18, 47], which *n* − 3 PUFA supplementation has a favorable effect on treating NAFLD [48], while there are also under-proof therapeutic approaches to the effect between *n* − 3 PUFAs and NAFLD [49].

Although our MR results do not provide sufficient genetic evidence for a causal relationship, several biological mechanisms have been proposed to explain how certain FFAs may influence NAFLD development. For example, SFAs such as palmitic acid may promote hepatic lipotoxicity, oxidative stress, and apoptosis [50, 51], while unsaturated fatty acids [52], particularly *n* − 3 PUFAs [53], have been shown to exert anti-inflammatory and insulin-sensitizing effects. These mechanisms may modulate hepatic fat accumulation, inflammation, and fibrosis—hallmarks of NAFLD progression [54–56]. The absence of causal associations in our MR findings does not preclude the possibility of context-specific or nonlinear effects, particularly under the conditions of metabolic stress or diet-induced dysregulation.

Most of the observations conduct the effect of FFAs by diet intervention, and effective preventive and therapeutic interventions for NAFLD necessitate a focus on lifestyle modifications. However, accurately quantifying habitual dietary fatty acid intake remains a significant challenge. Traditional dietary assessment methods are susceptible to various limitations, including measurement errors, recall bias associated with past dietary behaviors, increased participant burden during data collection, and social desirability bias where participants might under-report unhealthy dietary patterns [57]. High-carbohydrate diets are demonstrably associated with metabolic complications encompassing NAFLD [58]. This association stems from the fact that carbohydrates are a primary source for hepatic FFA production, particularly fructose and sucrose. In individuals with NAFLD, dietary sources can contribute up to 30% of FFA production, exceeding that of healthy individuals by a factor of six [59, 60]. However, hepatic FFA production is also influenced by the tightly regulated expression of enzymes involved in de novo lipogenesis (DNL), which exhibits compromised activity in NAFLD patients [60]. The limitation is to manage this growing public health concern [61]. While advancements in high-throughput metabolomics offer the potential to utilize circulating fatty acids as biomarkers of dietary intake, this approach is not without limitations. Certain medications, such as statins, and lifestyle factors, like alcohol consumption, can confound the interpretation of these biomarkers. For instance, studies have shown an association between statin and alcohol use with lower plasma linoleic acid levels [62]. These limitations highlight the complexities involved in accurately assessing dietary fatty acid intake in the context of NAFLD research. While FFAs are traditionally considered central to the development of NAFLD, emerging evidence suggests that dietary carbohydrates, particularly those that promote hepatic DNL, also play a significant role in the pathogenesis of the disease. Both FFAs and carbohydrates contribute to hepatic steatosis through distinct mechanisms, highlighting the importance of dietary interventions targeting both macronutrients in the management of NAFLD. Understanding the causal effects of FFAs would allow for the development of more targeted dietary recommendations for NAFLD prevention and management. Given the complexities surrounding dietary intake assessment and the limitations of traditional methods [61], it is a stable and appliable way to scale the association between genetic traits of FFAs and NAFLD. And genetically instrumented FFAs offer a robust approach to address confounding factors in observational studies of nutrition and attenuate measurement error associated with self-reported dietary intake [63]. These fourteen tightly related SNPs we chose to study are reported in different published studies [35–38] and are fully representative of the FFAs, and it is a lifelong signature for both populations and individuals. And epidemiological studies only focused on a specific type of FFAs, or it would be inevitable to consume too many resources to test individually; otherwise, if choosing one as a representative FFA, as we know, among SFAs, palmitic acid (16:0) exhibits the highest abundance. Similarly, oleic acid (18:1*n* − 9) is the most prevalent MUFA, while linoleic acid (LA; 18:2*n* − 6) holds the distinction of being the most frequent PUFA. [29–32], it would decrease the variety of FFAs. While MR analysis applied SNPs as instrumental variables, it is cost-efficient depending on large GWASs and case-control studies with extensive genotyping [64].

There are also limitations in the study. To minimize the bias from population structure, the participants were restricted to European ancestry for analysis. While this also narrows the effect of FFAs only to European ancestry, this result cannot reflect the situation in other ancestries. Another aspect is the catering characteristics among different age periods in the population, which is a key determinant of the metabolic rate. In addition, some of the IVs are shared by more than one FFA, such as rs174547 is shared by ALA, LA, and AA, and some IVs of FFAs are less than three, but as we already demonstrated previously, the reliability is also guaranteed in former published articles [35–38]. With more discoveries on human genetic resources, this insufficiency would be brought up to full strength. Moreover, while the discussion speculates on potential dietary implications, our study did not directly evaluate the relationship between dietary intake and circulating levels of individual fatty acids. The translation from intake to plasma concentration is influenced by multiple metabolic and physiological factors [65–67]. Therefore, dietary recommendations based on our findings should be interpreted with caution. Further research is needed to integrate genetic, dietary, and metabolomic data to clarify these links. Furthermore, although an LD clumping threshold of *R*^2^ < 0.001 is widely regarded as the strictest standard for selecting independent SNPs in MR studies, we used a slightly relaxed threshold of *R*^2^ < 0.005 in this analysis. This decision was made to retain a sufficient number of valid instruments, given the limited genome-wide significant SNPs available for several fatty acids.

## Figures and Tables

**Table 1:** Specific traits of FFA-related SNPs and the association on NAFLD.

Types of FA	FA	SNP	Chr	Nearby gene	EA	Beta	SE	P	*F* statistic	NAFLD		
										Beta adjust	Standard error	*p*_value
*n* − 3 PUFA	ALA	rs174547	11	FADS1	C	0.02	0.001	3.5E − 64	400.00	0.0132	0.0169	0.4338
	EPA	rs3798713	6	ELOVL2	C	0.04	0.005	1.9E − 12	64.00	0.0074	0.0163	0.6486
		rs174538	11	FADS1/C11orf10	G	0.08	0.005	5.4E − 58	256.00	−0.0241	0.0174	0.1661
	DPA	rs3734398	6	ELOVL2	C	0.04	0.003	9.7E − 43	177.78	0.0044	0.0163	0.7869
		rs174547	11	FADS1	T	0.08	0.003	3.8E − 154	711.11	−0.0132	0.0169	0.4338
	DHA	rs2236212	6	ELOVL2	G	0.11	0.014	1.3E − 15	61.73	−0.0071	0.0163	0.6649

*n* − 6 PUFA	LA	rs10740118	10	JMJD1C	G	0.25	0.05	8.1E − 09	25.00	−0.0388	0.0165	0.0189
		rs174547	11	FADS1	C	1.47	0.05	5E − 274	864.36	0.0132	0.0169	0.4338
		rs16966952	16	NTAN1	G	0.35	0.04	1.2E − 15	76.56	0.0122	0.0174	0.4845
	AA	rs174547	11	FADS1	T	1.69	0.02	3.3E − 971	7140.25	−0.0132	0.0169	0.4338
		rs16966952	16	NTAN1	G	0.2	0.03	2.4E − 10	44.44	0.0122	0.0174	0.4845

*n* − 7 MUFA	POA	rs6722456	2	RN7SKP93	G	0.05	0.009	4.1E − 08	30.86	−0.0928	0.0647	0.151
		rs603424	10	SCD/PKD2L1	G	0.03	0.004	5.7E − 15	56.25	0.00078	0.0225	0.9721
		rs11190604	10	HIF1AN	G	0.02	0.004	5.7E − 09	25.00	−0.0246	0.0201	0.2198
		rs102275	11	FADS1/2	C	0.02	0.003	6.6E − 13	44.44	0.0144	0.0168	0.3936

*n* − 9 MUFA	OA	rs102275	11	FADS1/2	C	0.23	0.02	2.2E − 32	132.25	0.0144	0.0168	0.3936

SFA	PA	rs2391388	1	ALG14	C	0.18	0.03	2.7E − 11	36.00	0.0025	0.0164	0.88
	SA	rs6675668	1	ALG14	G	0.17	0.02	2.2E − 18	72.25	−0.0028	0.0164	0.8608
		rs11119805	1	LPGAT1	T	0.17	0.03	2.8E − 09	32.11	−0.0395	0.0248	0.1119
		rs102275	11	FADS1/2	T	0.18	0.02	1.3E − 20	81.00	−0.0144	0.0168	0.3936

*Note:* Chr., chromosome; PUFA omega-3: ALA, α-linolenic acid (18:3*n* − 3); EPA, eicosapentaenoic acid (20:5*n* − 3); DPA, docosapentaenoic acid (22:5*n* − 3); DHA, docosahexaenoic acid (22:6*n* − 3); PUFA omega-6: LA, linoleic acid (18:2*n* − 6); AA, arachidonic acid (20:4*n* − 6); MUFA: POA, palmitoleic acid (16:1*n* − 7); OA, oleic acid (18:1*n* − 9); SFA: PA, palmitic acid (16:0); SA, stearic acid (18:0).

Abbreviation: EA, effect allele.

## Data Availability

The original contributions presented in the study are included in the article/Supporting Information, further inquiries can be directed to the corresponding authors, and all data used in this study are publicly available.

## Supporting Information

Table S1: Calculation of linkage disequilibrium of the selected SNP on chromosome 1.

Table S2: Calculation of linkage disequilibrium of the selected SNP on chromosome 10.

Table S3: Weighted median and MR-Egger analysis for genetic associations between exposures and NAFLD risk.
