# Supplementary material for: Causal Relationship Between Genetically Determined Free Fatty Acids and Nonalcoholic Fatty Liver Disease: A Mendelian Randomization Study
Source: Int J Endocrinol. 2025 Oct 7;2025:2181771. doi: 10.1155/ije/2181771 (PMC12520819; doi:10.1155/ije/2181771)
Supplement: Supporting Information — Additional supporting information can be found online in the Supporting Information section. [file 2181771.f1.docx]

**Causal relationship between genetically determined free fatty acids and nonalcoholic fatty liver disease: A Mendelian ran-domization study（supplementary Data）**

**Table S1 Calculation of linkage disequilibrium of the selected SNP on chromosome 1**

| SNP | rs6675668 | rs11119805 |
| --- | --- | --- |
| rs6675668 | 1.0 | 0.002 |
| rs11119805 | 0.002 | 1.0 |

The estimates of linkage disequilibrium are reported as r^2^ values in the table.

Population = (CEU) Utah Residents from North and West Europe; r^2^ < 0.005.

**Table S2 Calculation of linkage disequilibrium of the selected SNP on chromosome 10**

| SNP | rs603424 | rs11190604 |
| --- | --- | --- |
| rs603424 | 1.0 | 0.002 |
| rs11190604 | 0.002 | 1.0 |

The estimates of linkage disequilibrium are reported as r^2^ values in the table.

Population = (CEU) Utah Residents from North and West Europe; r^2^ < 0.005.

**Table S3 Weighted median and MR-Egger analysis for genetic associations between exposures and NAFLD risk**

| Method | Weighted median | MR-Egger | | MR-PRESSO |
| --- | --- | --- | --- | --- |
|  |  | Estimate | Intercept |  |
| LA | | | |  |
| Estimate (95% CI) | 0.01(-0.012, 0.032) | 0.026(-0.043, 0.096) | -0.024(-0.085, 0.038) |  |
| P value | 0.394 | 0.457 | 0.454 |  |
| POA | | | |  |
| Estimate (95% CI) | -0.056(-1.235, 1.124) | -1.687(-6.073, 2.669) | 0.035(-0.07, 0.141) |  |
| P value | 0.926 | 0.451 | 0.511 | 0.651 |
| SA | | | |  |
| Estimate (95% CI) | 0.0004(-0.062, 0.076) | -0.108(-5.275, 5.191) | 0.02(-0.918, 0.904) |  |
| P value | 0.376 | 0.987 | 0.988 |  |

**Table S4 MR-Steiger analysis for genetic associations between exposures and NAFLD risk**

| Type of FA | FA | MR-Steiger  Direction |
| --- | --- | --- |
| n-3 PUFA | ALA | TRUE |
|  | EPA | TRUE |
|  | DPA | TRUE |
|  | DHA | TRUE |
| n-6 PUFA | LA | TRUE |
|  | AA | TRUE |
| n-7 MUFA | POA | TRUE |
| n-9 MUFA | OA | TRUE |
| SFA | PA | TRUE |
|  | SA | TRUE |
